# Mechanisms of trauma at a rural hospital in Uganda

**DOI:** 10.4314/pamj.v7i1.69110

**Published:** 2010-10-16

**Authors:** Peter Hulme

**Affiliations:** 1 Trafford General Hospital, Moorside Rd, Davyhulme, Manchester M41 5SL

**Keywords:** Road Traffic Accidents, Trauma, Africa, Uganda, Trauma

## Abstract

**Introduction:**

Trauma is an increasing cause of mortality worldwide with road traffic accidents (RTAs) causing 1.3 million deaths annually with 90% of this mortality occurring in low and middle income countries. The rise in trauma deaths has been neglected with infectious diseases taking precedence. More research needs to be conducted in resource poor countries to establish the main causes of trauma and find better solutions to the rising trend in mortality. Much of the trauma research in resource poor countries has focused on urban areas. This study aims to find the leading causes of trauma at a rural Ugandan hospital.

**Methods:**

A retrospective case note review was performed on all adult patients admitted to Kuluva Hospital with trauma related injuries in 2007. Kuluva Hospital is a rural 250 bed hospital in North-West Uganda.

**Results:**

490 trauma patients were admitted in 2007 accounting for 9.4% of admissions. 70.2% (n=344) were males and 29.8% (n=146) were females. The mean age of patients was 31.3 years and the mean length of stay was 7.4 days. In 2007 9 patients died following trauma, 6 from RTAs, 2 from burns and one after an assault. RTAs were the leading cause of trauma with 64.2% of admissions (n=315), followed by assaults with 16.5% (n=81) of admissions. Soft tissue injuries with 28.4% (n=149) and lacerations with 27.3% (n=143) were the most common diagnoses after trauma with fractures making up 18.7% of injuries (n=99).

**Conclusion:**

RTAs were an important cause of morbidity and mortality in a rural Ugandan hospital as they also are in urban areas. Low cost initiatives to reduce speed, prevent alcohol impaired driving, improve public education and wider access to high quality trauma care are vital to reducing the mortality and morbidity caused by RTAs in Africa.

## Introduction

Trauma and in particular Road Traffic Accidents (RTAs) are a major cause of mortality and morbidity worldwide. The World Health Organisation (WHO) estimates that RTAs are the ninth highest cause of death accounting for 1.3 million deaths annually with this figure set to rise making RTAs the fifth leading cause of worldwide death by 2030 [1]. More than 90% of these deaths occur in low and middle income countries with young people, especially males disproportionately affected [2,3]. Deaths due to trauma and RTAs are rapidly overtaking infectious diseases as the primary cause of morbidity and untimely mortality worldwide and in Africa cause the loss of more life years than HIV and malaria combined [4,5]. However, whilst infectious diseases such as HIV, malaria and TB receive much international attention and funding, trauma has been relatively ignored by public health bodies [4]. The rising morbidity and mortality of trauma cannot be ignored much longer as RTAs are so often preventable and the means to exact change readily at hand [6].

To help us find better solutions to the problem of rising trauma deaths, we need to better understand the underlying causes of trauma particularly with research focused in low income countries.

Most of the trauma research in resource poor countries has focused on urban areas. This paper aims to assess the causes and pattern of injuries sustained over one year at a rural mission hospital in North-West Uganda.

## Methods

A retrospective case not review was conducted and the notes of all adult patients admitted to Kuluva Hospital in 2007 with trauma related injuries were assessed. For the purpose of this study adults were classified as patients aged 16 years and over. Due to incomplete notes 12 patients were excluded. The following observations were recorded: age and gender of the patient, the mechanism of injury, the injury sustained and length of stay. Kuluva Hospital is a 250 bed mission hospital set in rural North-West Uganda 9km from the town of Arua where the regional referral hospital is located. It is close to the borders of Congo and Sudan and receives patients from both these countries. The hospital is situated by a good quality tarmac road which is the major route to Kampala. The main type of transport is by motorcycle taxi, bicycles and cars.

## Results

In 2007, 502 patients were admitted to Kuluva Hospital with trauma related injuries. The hospital overall admitted 5358 adult patients to the medical, surgical and maternity wards with trauma accounting for 9.4% these admissions. Of the 490 trauma patients with complete notes 70.2 % (n=344) were males and 29.8% (n=146) were females. The mean age of the patients who had documented ages (n=375) was 31.3 years and the median age was 28 years. The mean length of stay was 7.4 days. Nine patients died (males n=8, females n=1) and of these 6 died in RTAs, 2 died following burns and one died in an assault. Of the remaining patients 457 were discharged, 4 ran away and 19 were referred to another hospital. The mechanism of trauma injuries was varied as shown in table 1 with RTAs being the most common mechanism of injury with increased frequency in males (76.5%, n=241) compared to females (23.5%, n=74). The mechanisms of RTAs are shown in table 2.

The injuries sustained from all mechanisms of trauma are shown in table 3 with the common injuries being soft tissue injuries and lacerations. Fractures were responsible for 18.7% of injuries with a number of patients having sustained multiple limb fractures.

Assaults were the second commonest cause of admission (16.5%, n=81) and accounted for one death (Table 4). The frequency of assaults was higher in males (61.2%, n=50) compared to females (38.2%, n=31) and the association with alcohol was documented in 5 cases. Where the Page number not for citation purposes 3 assailant was documented it was most commonly a stranger, family member or spouse with women particularly likely to be injured by their husbands.

## Discussion

The study highlights a number of important points in relation to causes of trauma presenting to a rural hospital in Uganda. Chiefly it showed that RTAs were the major cause of hospitalisation and death. Efforts to reduce trauma mortality in resource poor countries should target the central causes of accidents and implement low cost methods to reduce the frequency of injuries.

Alcohol remains the most commonly abused drug in trauma victims although the use of other drugs is increasing [7]. Although this study didn’t specifically look at the relationship of alcohol and trauma it is well known that alcohol consumption increases the risk of RTAs and especially fatal crashes [8]. A recent Cochrane review concluded that police patrols aimed at stopping the adverse consequences of alcohol on driving reduce the incidence and mortality of trauma [8].

Education of hospital staff can improve trauma care as has been demonstrated in Ghana and Tanzania [9-10]. A novel approach to improving prehospital trauma care has been trialled in Tanzania where lorry drivers in areas with no emergency transport were given basic trauma care training [11].

Motorcycle users form a high proportion of those killed or injured in RTAs with head injuries being a common feature [12]. Despite this fact many motorcycle users in resource poor countries don’t wear helmets, even though this is against the law. Evidence shows that helmet use reduces the risk of mortality and head injury in motorcycle riders who crash [12] and that the benefits of wearing helmets also applies to bicycle users [13]. Efforts to reduce injuries should focus on promoting helmet usage and ensuring police officers enforce existing laws.

High speed is a key risk factor for RTAs [14]. Both speed enforcement detection devices and speed bumps have both been shown to be successful ways to reduce RTAs [15-16]. A study in Uganda showed that increased police patrols to stop dangerous driving can be a low cost means to reduce RTA mortality [17]. Sadly for Africa though, the combination of low enforcement levels, police corruption and low levels of public awareness and compliance with speed restriction limits lessens the impact of speed limits [18]. Studies have shown that seat belts can reduce fatalities by 50% and serious injury by 55%. Unfortunately anecdotal evidence from developing countries suggests that only half of cars have functional seatbelts and when they are present they aren’t used [7] highlighting a problem with compliance.

In Africa motor vehicles are often old and poorly maintained. Governments have tried to stop importation of older vehicles but this has proved difficult to enforce [7,18]. Interventions aimed at removing dangerous vehicles from the roads and maintaining the quality of vehicles are urgently needed as this area has been largely ignored and may reduce the frequency of accidents.

Assaults were the second most common cause of injury in this study. Assaults and interpersonal violence are a growing worldwide problem particularly in resource poor settings where the WHO says 90% of interpersonal violence occurs [19-20]. As with RTAs alcohol is also believed to play a significant part in interpersonal violence [21-22]. In this study it was shown that women were more likely than men to be assaulted by their spouse, which was similar to the findings in a recent study from Kenya [23]. Due to gender inequalities in Uganda the number of women presenting to hospital after an assault may well be a low estimate with women being unable to seek medical attention due to cultural or financial reasons.

The true incidence of trauma relies on patients presenting to hospitals. Many victims of trauma may die at the scene of the accident or preferentially seek the care of traditional healers meaning the true scale of the problem is difficult to calculate. Due to this the numbers of deaths Page number not for citation purposes 4 and patients represented in this study are likely to be a low estimate of the actual number of trauma cases occurring in the hospital locality. Also patients may have presented to the district referral hospital in Arua which would also have affected patient numbers.

Many people in this area don’t know their exact age so some ages were described as ‘adult’ therefore affecting the average age. In 209 cases the patient was described as being ‘involved in an RTA’ and so due to the retrospective nature of the study we don’t know how the patient actually came to be injured in terms of if they were a pedestrian, cyclist, driver etc. The same applies to the way a patient was injured in an assault which explains why in 30 cases we don’t know who caused the assault.

## Conclusion

Trauma is a rising cause of mortality worldwide with RTAs causing the majority of injuries. As in urban hospitals RTAs cause significant morbidity and mortality in rural areas. Greater global awareness, more research and renewed emphasis needs to be placed on trauma as a means to reduce this mortality and morbidity. Much of the global burden of trauma is preventable and so initiatives to reduce speed, reduce alcohol impaired driving, improve public education and improved access to high quality trauma care are vital to reducing the mortality and morbidity caused by RTAs in Africa.

## Acknowledgements

Many thanks to Professor Geoff Gill at the Liverpool School of Tropical Medicine and Hygiene for help and advice on preparing the original manuscript.

## Figures and Tables

**Table 1:** Mechanism of trauma injury at Kuluva Hospital (Uganda, 2007)

**Mechanism of injury**	**No. of patients**	**% of trauma admissions**
Road traffic accident	315	64.2%
Assault	81	16.5%
Fall	33	6.7%
Snake bites	18	3.6%
Burns	12	2.4%
Collapsed wall	9	1.8%
Injured by tools	9	1.8%
Animal bite	4	0.8%
Fall from tree	4	0.8%
Attempted hanging	2	0.4%
Miscellaneous	3	0.6%

**Table 2:** The mechanisms of road traffic accidents at Kuluva Hospital (Uganda, 2007)

**Mechanism of RTA**	**No. of patients**
Not specified	209
Fall off motorcycle	48
Fall off bicycle	18
Pedestrian hit by vehicle	14
Pedestrian hit by motorbike	11
Pedestrian hit by bicycle	5
Overturned vehicle	4
Fall off moving vehicle	4
Hit by motorbike whilst on bicycle	2

**Table 3:** Injuries sustained in patients with trauma at Kuluva Hospital (Uganda, 2007)

**Injury sustained**	**No. of patients**	**% of injuries**
Soft tissue injury	149	28.4%
Laceration	143	27.3%
Minor head injury	63	12.0%
Major lower limb fracture	43	8.2%
Minor fracture	24	4.5%
Major upper limb fracture	19	3.6%
Snake bite wound	18	3.4%
Burns	12	2.2%
Intracerebral haemorrhage	10	1.9%
Skull fracture	9	1.7%
Joint dislocation	8	1.5%
Cervical spine fracture	4	0.7%
Perforation of abdominal organ	3	0.5%
Miscellaneous injury	18	3.4%

**Table 4:** Assailant of patients with trauma from assaults at Kuluva Hospital (Uganda, 2007)

**Assailant**	**Males (Number)**	**Females (Number)**	**Total**
Not documented	17	13	30
Stranger	16	3	19
Family member	9	3	12
Spouse	1	11	12
Mentally ill person	2	1	3
Robbers	2	0	2
Mob	3	0	3

## References

[1] The Global Burden of Disease 2004 Update.. World Health Organisation 2004.

[2] Milestones in International Road Safety. World Health Day 2004 and Beyond. (2004).

[3] Bowley D (2002). The malignant epidemic - Changing patterns of trauma.. South African Medical Journal..

[4] Meel BL (2004). Incidence and patterns of violent and/or traumatic deaths between 1993 and 1999 in the Transkei region of South Africa.. J Trauma..

[5] Bergman, Simon (2008). Assessing the Impact of the Trauma Team Training Program in Tanzania.. J Trauma..

[6] Hazen A, Ehiri J (2006). Road traffic injuries: hidden epidemic in less developed countries.. J Natl Med Assoc..

[7] Peden M (2000). Substance abuse and trauma in Cape Town.. South African Medical Journal..

[8] Goss C (2008). Increased police patrols for preventing alcohol-impaired driving.. Cochrane Database Syst Rev..

[9] Bergman S (2008). Assessing the impact of trauma team training in Tanzania.. J Trauma..

[10] Mock C (2005). The development of continuing education for trauma care in an African nation.. Injury..

[11] Mock CN (2002). Improvements in prehospital trauma care in an African country with no formal Emergency Medical Services.J Trauma..

[12] Liu B (2008). Helmets for preventing injury in motorcycle riders.. Cochrane Database Syst Rev..

[13] Thompson D (2000). Helmets for preventing head and facial injuries in bicyclists.. Cochrane Database Syst Rev..

[14] Forjuoh S (2003). Traffic-related injury prevention interventions for low-income countries.. Inj Control Saf Promot..

[15] Wilson C (2006). Speed enforcement detection devices for preventing road traffic injuries.. Cochrane Database Syst Rev..

[16] Afukaar F (2003). Speed control in developing countries: issues, challenges and opportunities in reducing road traffic injuries.. Inj Control Saf Promot..

[17] Bishai D (2008). Cost-effectiveness of traffic enforcement: case study from Uganda.. Injury Prevention..

[18] Lagarde E (2007). Road traffic injury is an escalating burden in Africa and deserves proportionate research efforts.. PLoS Med..

[19] Rutherford A, Zwi AB, Grove NJ (2007). Violence: a priority for public health? (part 2).. J Epidemiol Community Health..

[20] Dahlberg LL, Krug EG Violence: a global public health problem.. In: Krug EG,Dahlberg LL, Mercy JA, et al, eds. World report on violence and health.

[21] Macdonald (2005). The criteria for causation of alcohol in violent injuries based on emergency room data from six countries.. Addict Behav..

[22] (2006). WHO. Policy briefing: interpersonal violence and alcohol.. Geneva: World Health Organization.

[23] Ranney M (2009). Injuries from interpersonal violence presenting to a rural health center in Western Kenya: characteristics and correlates.. Injury Prevention..

